# Bilateral High Trifurcation of the Common Carotid Artery and Variable Emergence of the Lower Branches of the External Carotid Artery: A Cadaveric Case Report

**DOI:** 10.7759/cureus.27657

**Published:** 2022-08-03

**Authors:** Amit K Shreevastava, Rajat S Das, Tarun P Maheshwari, Balkund K Damodhar

**Affiliations:** 1 Anatomy, All India Institute of Medical Sciences, Raebareli, IND

**Keywords:** remodeling, linguofacial trunk, external carotid artery, carotid termination, common carotid artery

## Abstract

Trifurcation of the common carotid artery in the neck region is a rare anatomical variation. In the present study, we reported a rare case having the combination of anomalies of the bilateral high common carotid arteries trifurcation and variable origin of lower branches of the external carotid artery during routine dissection of the head and neck region of a 60-year-old male cadaver in the Department of Anatomy. Both on the left and right sides of the neck region, the common carotid artery gave off three terminal branches: internal carotid artery, external carotid artery, and ascending pharyngeal arteries. Further, we also observed the presence of bilateral linguofacial trunks (common arterial trunks) that emerged from the external carotid arteries and also the left superior thyroid artery that originated directly from the left common carotid artery. Even though the embryogenesis of the variable origin of such arterial trunks is not apparent, it is very indispensable to have sound knowledge and better comprehension of the accurate anatomical architecture of such a rare combination of carotid arterial system anomalies for correct interpretation of the vascular imaging that pave the pathway for successful execution of surgical interventions in the neck region because of its utmost clinical implication.

## Introduction

The right and the left common carotid artery and its branches are the vital sources of arterial vascularisation of the head and neck and region. The right common carotid artery emerges from the brachiocephalic trunk and the left common carotid artery comes off the arch of the aorta [1]. Normally, on both sides, the common carotid artery (CCA) bifurcates into an internal carotid artery (ICA) and an external carotid artery (ECA) in the carotid triangle at the level of the upper border of the thyroid cartilage anteriorly which corresponds with the intervertebral disc between C3-C4 cervical vertebrae posteriorly. The ECA normally gives six branches in the neck region which are namely (arranged according to the ascending order of their site of origin): superior thyroid artery, ascending pharyngeal artery, lingual artery, facial artery, occipital artery, and posterior auricular artery, and finally terminates behind the neck of the mandible into the max­illary and superficial temporal arteries [1]. Normally, the superior thyroid artery (STA), the lingual artery (LA), and the facial artery (FA) arise from the ventral surface, the ascending pharyngeal artery (APA) arises from the medial surface, the occipital artery (OA) and the posterior auricular artery (PAA) arise from the posterior surface of the ECA [1]. The neck and face regions are predominantly nourished by the ECA and its branches. The ICA does not provide any arterial branch in the neck area and enters the skull through the carotid canal and nourishes the brain [1].

In the present cadaveric case, we reported a rare combination of bilateral high trifurcation of the CCA which terminated as the ICA, the ECA, and the APA, the bilateral origin of the linguofacial trunk (LFT) from both the ECA and variable origin of the left STA from the left CCA which has not been earlier documented in the available articles. 

The objective of our study is to share the recent modifications in the anatomical architecture of the carotid arterial system, probable explanation of the embryological basis of these anomalies, and the morphometric status of the arterial trunks for correct diagnostic and surgical approach for better health care management.

## Case presentation

**Figure 1:**
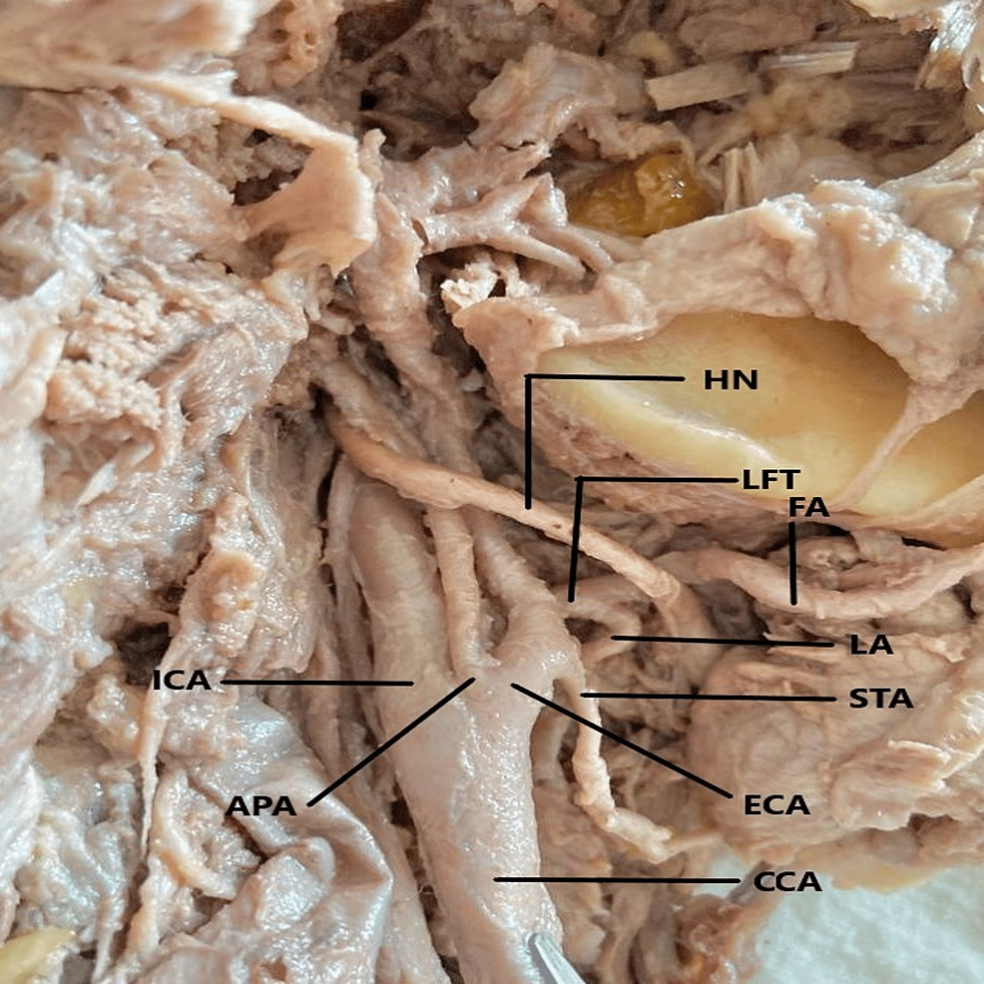
Trifurcation of the right common carotid artery and branching pattern of the external carotid artery. CCA - common carotid art.; ECA - external carotid art.; ICA - internal carotid art.; APA - ascending pharyngeal art.; STA - superior thyroid art.; LFT - linguofacial trunk; FA - facial art.; LA - lingual art.; HN - hypoglossal nerve.

**Figure 2:**
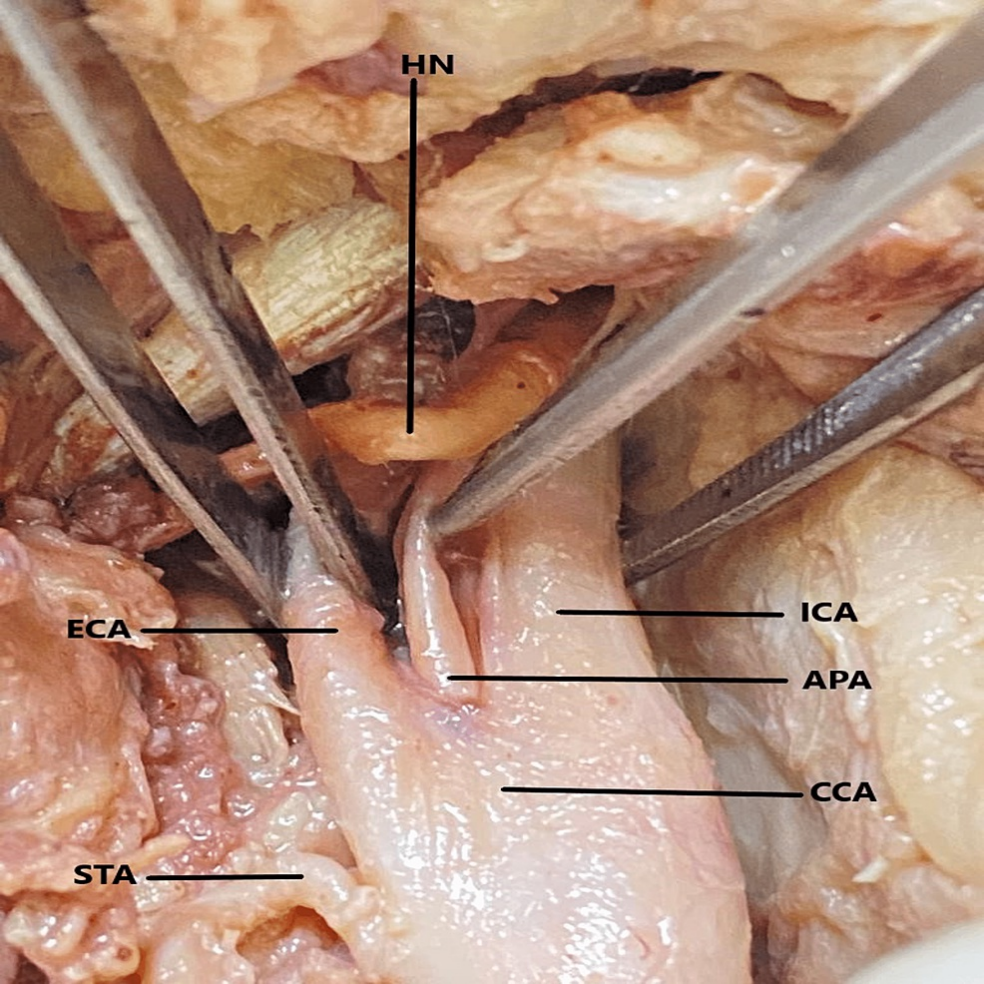
Trifurcation of the left common carotid artery and the branching pattern of the external carotid artery. CCA - common carotid art.; ECA - external carotid art.; ICA - internal carotid art.; APA - ascending pharyngeal art.; STA - superior thyroid art.; HN - hypoglossal nerve.

**Figure 3:**
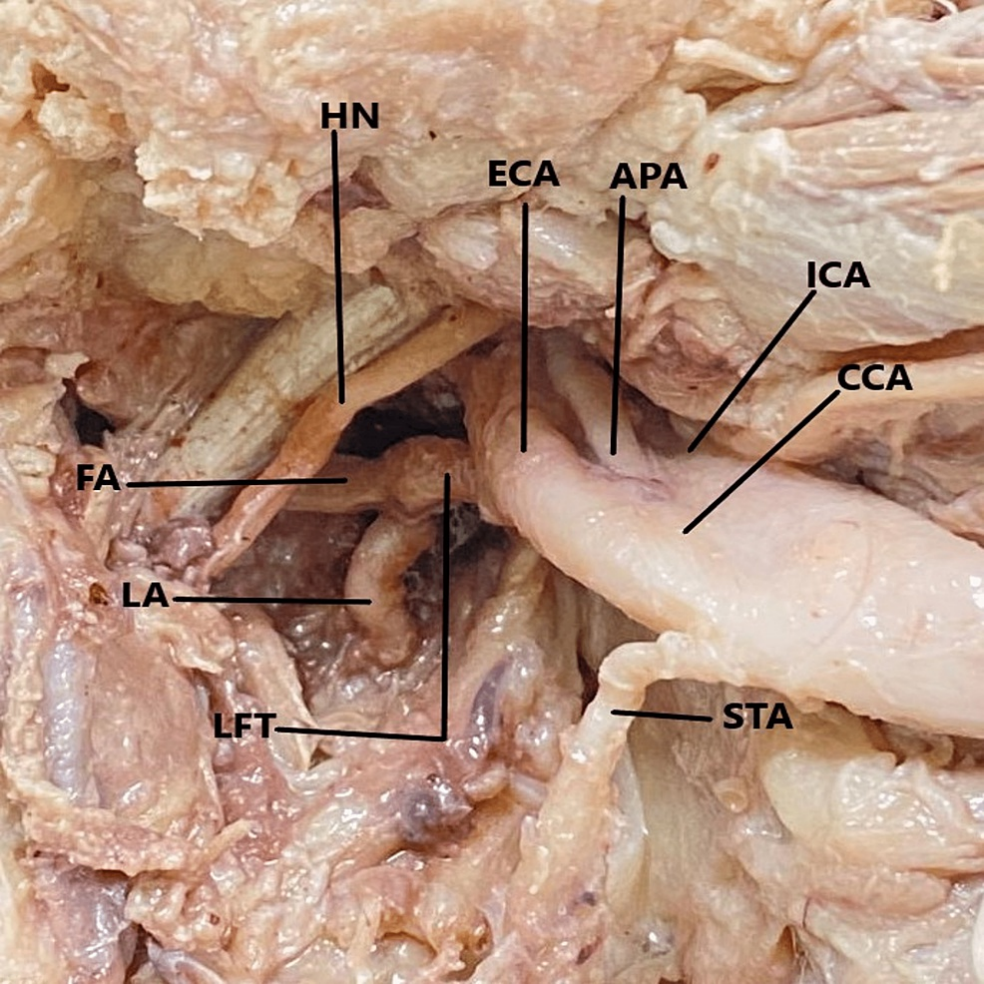
The exposure of the deep (internal) surface of the left common carotid artery and its terminating branches as they are turned backward. CCA - common carotid art.; ECA - external carotid art.; ICA - internal carotid art.; APA - ascending pharyngeal art.; STA - superior thyroid art.; LFT - linguofacial trunk; FA - facial art.; LA - lingual art.; HN - hypoglossal nerve.

During scheduled dissection of the neck area in an embalmed 60-year-old male cadaver, for M.B.B.S. students in the Department of Anatomy, during the year 2021-2022, it was observed that both right and the left CCA terminated into the ICA, ECA, and the APA. The anatomical position of the APA was in between the ICA and the ECA. The CCA was found to follow a straight course from the site of emergence to the point of termination. On both sides, the termination of the CCA was higher up corresponding with the greater cornu of the hyoid bone at the level of body of C3 (Figures 1, 2, 3). 

On further exploration of the carotid arterial system, it was noticed that on both sides of the neck, the lingual and facial arteries emerged from a common arterial trunk called the linguofacial trunk (LFT). On the right side (Figure 1), the LFT came off from the ventral aspect of the ECA while on the left side (Figures 2, 3), it originated from the deep (internal) aspect of the ECA. The LFT is further divided into the FA and the LA on each side. The STA arose from the anterior surface of the ECA on the right side but came off from the medial aspect of the CCA on the left side. The remaining branches of the ECA originated from their usual site. The hypoglossal nerve passes superficially, as much proximal to the carotid termination and LFT division site while maintaining the usual relationship to the carotid sheath.

The morphometric details of the CCA, ICA, ECA, LFT, APA, STA, LA, and FA were calculated with the use of a good quality digital vernier caliper (Freemans FDC 150; FMI Ltd., Doraha, India). During the measurement, no manual force had been applied to the arteries and in situ anatomical position of the arteries was maintained. The diameter of the CCA was determined at 1 cm before termination and for ICA just distal to carotid sinus. The diameter of the remaining arteries/trunks was determined at 2 mm from their point of origin. On the right side, the emergence points of the STA and the LFT were at 4.6 mm and 9.8 mm from the carotid termination respectively. Similarly, on the left side, the arising points of the STA and the LFT were 5 mm and 9.2 mm from the carotid termination respectively. The length of the right and left LFT was 3.8 mm and 3.9 mm respectively.

## Discussion

The genesis of the blood vascular system is a very complex affair [2]. The blood vessels develop mainly by three mechanisms: (1) vasculogenesis during which angioblasts coalesce to give rise to an initial vessel network; (2) angiogenesis where new vessels come out as sprouting manner from the existing vessels and abruptly go through extensive remodeling; (3) vascular intussusception (non-sprouting angiogenesis), where existing vessels split or fuse and give rise to extra vessels. The development of the head, neck, and face regions is too complicated where the vascular remodeling process is very dynamic that may transform the aortic arches concerned with the region [2].

In the present instance, we hypothesized that high termination of the CCA and anomalous emergence of the lower branches of the ECA may be due to (1) The disproportionate cranial extension of the truncus arteriosus also causes an upward shift of the aortic arches leading to a higher origin of the CCA which ultimately give rise to higher CCA termination; (2) After the formation of the CCA and the ICA, the ECA, later on, emerges in sprouting mode from the CCA which arises from the third aortic arch. Since the ECA comes out in a sprouting manner, it goes through extensive remodeling and also takes a contribution from the proximal remnants of the first and second aortic arches and finally gives rise to its arterial branches; (3) Due to high CCA termination, the branches of the ECA get less space in the neck region and migrate caudally. This phenomenon may be responsible for the extensive remodeling of the lower arterial branches of the ECA giving rise to a common arterial trunk and emergence from the variable site which may be from the CCA and/or carotid termination. 

Normally, the CCA bifurcates into the ICA and the ECA at the anterior upper border of the thyroid cartilage and the intervertebral disc level between the C3-C4 cervical vertebrae posteriorly [1]. Termination of CCA is called high when it divides above the level of the superior border of the thyroid cartilage anteriorly and the level of the intervertebral disc between the C3-C4 cervical vertebral level [3]. Several articles have mentioned the variable height of the CCA termination [3-8]. In a recent radiological study, the variable levels of carotid termination were observed at the level of the intervertebral disc of the C3-C4 vertebra at the frequency of 29.6%, with 79.6% between the vertebral bodies of C3 and C4. Another 9.8% had a high CCA termination above cervical vertebral body C3 and 10.5% had a low CCA termination below C4 vertebral body [4]. In the present case the carotid termination has been observed at the level of the body of C3.

In the case of high CCA termination, the hypoglossal nerve comes very close to the termination and thus becomes vulnerable to injury during various types of surgical interventions like carotid endarterectomy, carotid stenting, and radical neck dissection, fasciocutaneous flaps of the neck, etc. [6-8]. Some authors also described the reversed position of the ICA and the ECA in 1.7% - 7.5% of cases [8,9]. If the surgeon is unaware of this crucial arterial variation and during operative procedure clamps the ICA in place of the ECA due to lack of this vital knowledge results in grave consequences including the paralysis of the patient [6,8].

Earlier studies have documented the anomalous emerging architecture of the lower arterial branches of the ECA [4,9]. In a radiographic study, it was mentioned that the APA originated from carotid termination in 6.5% of cases [3]. On the other hand, in a cadaveric study, the APA came off the carotid termination in 5% of cases [10]. During the interventional endovascular procedure at the carotid termination, the clinician must take extra precautions when the APA comes off from the CCA termination site in the case of carotid trifurcation because the cranial nerves IX, X, XI, and XII are supplied by the APA and if it is damaged or thrombosed then paralysis of the lower cranial nerves will happen [10].

The STA is considered the most unpredictable arterial branch of the ECA related to its origin and course [4]. In a previous radiological study, the emergence site of the STA was mentioned as 55.9% from the ECA, 24.3% from the carotid termination, and 18.4% from the CCA. The site of emergence of the STA was at a mean distance of 6.7 mm above the CB when originated from the ECA, and 7.5 mm below when arose from the CCA [4]. In the present study, the distance between the CCA termination and the point of emergence of the STA was 4.6 mm and 5 mm when it came off from the ECA and the CCA respectively. The STA's unpredictable origin and variable course may increase the chances of external laryngeal nerve injury during neck surgery. During the surgery of the upper pole of the thyroid gland, if the oozing of blood goes on even after the proper ligation of the ECA, the operating surgeon should consider the emergence of the STA either from the CCA or carotid termination [11].

In the present observation, the LA and the FA arose from a common arterial trunk called the LFT which originated from the ECA. The occurrence of the LFT was documented in 7.5% of cases by Ozgur et al. [12], while Fazan et al. reported LFT to be present in 20% of cases on the right side and 24% on the left side [13]. They also reported the incidence of bilateral LFT in 4.9% of cases. Lucey et al. documented the incidence of LFT at 20% [14]. In the present report, the site of origin of the LFT was found at 9.8 mm and 9.2 mm from the CCA termination on the right and left sides respectively. The length of the right and left LFT before splitting into the LA and the FA was 3.8 mm and 3.9 mm respectively. Troupis et al. in their study mentioned that the site of emergence of the LFT was at 7.6 mm and 6.8 mm from carotid termination and the length of the LFT before division into the LA and the FA was at 3.1 mm and 3.4 mm on the right and the left sides respectively [15]. Gonzalez et al. stated that the walls of the LA and the FA become very thick and tough when they came off from the LFT and that may be the probable reason for developing pseudoaneurysms in the ECA after catheterization procedures [16].

It has been observed that people having carotid system vascular anomalies are generally asymptomatic and revealed incidentally either during vascular imaging or at the time of emergency surgery in the neck territory [5].

In the following table (Table 1), we have also documented the comparative morphometric values of the diameter of the CCA and its branches in the current and earlier studies [4,9,10,13]. However, those studies mentioned the mean diameter of the right and left arteries of the carotid arterial system.

**Table 1:** Comparative morphometric values of the diameter of the common carotid artery (CCA) and its branches in the current and earlier studies

Name of the artery	Past study	Present case report	
	Mean Arterial diameter (mm)	Arterial diameter (mm)	
		Right side	Left side
Common carotid artery [9]	8.1± 2.24	8.4	8.3
Internal carotid artery [9]	6.1 ± 1.3	6.8	6.4
External carotid artery [9]	6.6 ± 1.3	5.4	5.2
Linguofacial trunk [13]	2.4 ± 0.02	3.5	3.2
Ascending pharyngeal artery [10]	1.5 ± 0.25	2.2	1.8
Superior thyroid artery [4]	1.7 ± 0.46	1.8	1.8
Lingual artery [4]	1.95 ± 0.57	2.1	2.2
Facial artery [4]	2.45 ± 0.66	2.8	2.6

## Conclusions

It is very difficult to precisely explain the embryological basis of the appearance of the variable arterial architecture of the carotid arterial system in which the nature of genesis of the ECA and its branches are the most variable due to increased remodeling. We understand that the development is very dynamic in the regions of the head, neck, and face, which would result in the corresponding changes in the hemodynamic behavior of the blood vessels, and lead to the possibilities of variable arterial branching manifestation in these regions. We conclude that if the termination of the CCA will be higher, then it will increase the possibilities of the emergence of the lower branches of the ECA from the CCA or the carotid termination, and also enhances the formation of the common arterial trunks from the former.

We appreciate that such type of rare combinations of variable arterial design of the carotid system will be interesting from the academic and surgical point of view as well. It would provide useful information during head and neck surgeries. This piece of information about the variable arterial architecture of the common carotid artery and its branches would also be of help to interventional radiologists. We expect the surgeons would be more vigilant during various head and neck surgeries and intervention procedures to minimize untoward postprocedural complications.
